# Meaning Making for Psychological Adjustment and Quality of Life in Older Long-Term Breast Cancer Survivors

**DOI:** 10.3389/fpsyg.2021.734198

**Published:** 2021-09-28

**Authors:** Marianne Nilsen, Ragna Stalsberg, Kari Sand, Gørill Haugan, Randi Johansen Reidunsdatter

**Affiliations:** ^1^Department of Social Work, Norwegian University of Science and Technology (NTNU), Trondheim, Norway; ^2^Department of Circulation and Medical Imaging, Norwegian University of Science and Technology (NTNU), Trondheim, Norway; ^3^Department of Health Research, SINTEF Digital, Trondheim, Norway; ^4^Department of Public Health and Nursing, Norwegian University of Science and Technology (NTNU), Trondheim, Norway

**Keywords:** survivorship, breast cancer, meaning making, quality of life, coping, well-being

## Abstract

**Objectives:** This study aims to explore in depth the meaning and meaning discrepancies among older Norwegian breast cancer survivors in light of the meaning making model by Park (2013).

**Design:** We utilized a qualitative design collecting data using semi-structured interviews of 23 elderly breast cancer survivors 7–8 years after treatment. The interviews followed an interview guide structured along three main themes: “everyday life activities,” “follow-up-care experiences” and “health status and QoL.”

**Results:** Several health problems were reported by the women in the aftermaths of the disease, such as sleeping problems, pain, and fatigue—including cognitive and emotional impairments. Meaning discrepancies were concentrated on six main themes: shifting perspectives and priorities, growing sense of autonomy, widening the limits of normality, dissociating oneself from the disease, embracing alternative health services, and feeling lucky. The women engaged in a wide range of coping techniques as efforts to change global meaning, and to develop a more positive view on the cancer experience. Common coping efforts across the six main themes were social comparison, denial, positive reappraisal, problem-focused coping, and revaluing ordinary events.

**Conclusion:** Many cancer patients report on unmet needs for help with their meaning making, and the facilitation of meaning making processes is rarely included in the follow-up care of cancer survivors. The findings of the present study may help health care professionals provide care for women who have experienced breast cancer. The concrete knowledge of common coping efforts in the meaning making process may contribute to the development of future interventions and for gaining a deeper understanding for older survivors of breast cancer.

## Introduction

As breast-cancer treatments have become more advanced, more than 90% of women experiencing breast cancer enters long-term survivorship (>5 years after diagnosis) (Ferlay et al., 2018). From 1989 to 2006, the breast cancer mortality rate among middle aged and older women decreased with more than 20% across many European countries, including Norway (Autier et al., 2010). Struggles with somatic and mental late effects after treatment are well documented (Bower et al., 2005; Koch et al., 2013; Fielding and Lam, 2014; Kenne Sarenmalm et al., 2014; Sekse et al., 2019). Accordingly, a large proportion of women will enter retirement with complex health care needs. Estimates based on the United States population conclude that by 2040, 73% of all cancer survivors of any type will be older than 65 years (Bluethmann et al., 2016). Moreover, living beyond breast cancer as a life-threatening illness may raise fears of recurrence and existential concerns, accompanied by increased vulnerability; all which challenge people’s perceived meaning in life (Gall and Cornblat, 2002; Heine et al., 2006). Hence, meaning making processes to adjust to adversity will be initiated. In general, to perceive one’s life as meaningful is crucial to people’s mental and existential wellbeing and health, and perceived meaning in life is found to be a vital health-promoting resource among vulnerable populations such as cancer patients and survivors (Park et al., 2008; Park and George, 2013; van der Spek et al., 2017; Haugan and Dezutter, 2021). Furthermore, from a developmental perspective, with age follows a need to look back on life, rather than to the future, and create meaning from all the experiences of the past (Erikson, 1980). The importance of meaning making for older individuals are supported by several studies linking meaning making to QoL and well-being for this population (Drageset et al., 2017; Haugan et al., 2020; Haugan and Dezutter, 2021). Consequently, relevant supportive health care interventions addressing not only the comorbidity burden among older long-term breast cancer survivors, but also promoting existential and mental wellbeing will be even more important in the years to come (Winger et al., 2020). However, studies addressing perceived meaning and meaning making as a health-promoting resource among long-term breast cancer survivors are scarce (Schroevers et al., 2004; van der Spek et al., 2013). To improve older breast cancer survivors’ health and wellbeing, knowledge about meaning making processes in this population is important (Haugan and Eriksson, 2021; Lopez and Klainin-Yobas, 2021). Therefore, this study addresses this knowledge gap, exploring in depth the meaning making among older long-term breast cancer survivors.

### Meaning Making and Psychological Adjustment Among Cancer Patients

Breast cancer is a life-threatening disease in which the patients lack influence on the outcomes of the illness. In such low-control situations, meaning making—the ability to transform meaning—is found to be a highly adaptive coping strategy for psychological adjustment (Park et al., 2001, 2008). Perceived meaning in life is understood as expectations or schemes, enabling people to have control of oneself and one’s environment and thus feeling secure while facing difficulties (Proulx and Inzlicht, 2012). The meaning making model developed by Park (2013) separates meaning in two levels:(1) *global meaning*, i.e., general understanding of how the universe works, self-identity, control, justice, God, goals, sense of meaning/purpose, and (2) *situational meaning*, i.e., meaning of specific events such as an illness (Park, 2013).

A strong sense of *global meaning*, across all domains (i.e., personal control, purpose in life) relates to increased psychological health and quality of life (QoL) among cancer patients (Tomich and Helgeson, 2002; Laubmeier et al., 2004; Winger et al., 2016; Majerníková and Obročníková, 2017). According to the meaning making model, *situational meaning* is the specific evaluations of the cancer illness, such as viewing the event as (un)controllable and (un)fair, and to what degree it violates the persons own goals (Park and Gutierrez, 2013). In comparison to global meaning, situational meaning seems to be a weaker and less consistent predictor of QoL (Sherman et al., 2010). A longitudinal study of breast cancers survivors (median of 24 months since diagnosis) reported that “having found illness-specific meaning” was not related to either distress, health related QoL, or breast cancer problems 4 months after being diagnosed (Sherman et al., 2010). Searching for (as opposed to having found meaning) illness-specific meaning, on the other hand, predicted poorer adaptation in terms of increased distress and poor QoL (Sherman et al., 2010). The relationship between searching for meaning in the illness and less adaptive outcomes may be explained by rumination—the tendency to engage in repetitive, aversive, and uncontrollable thoughts such as asking why the illness happened to me. As a breast cancer patient moves beyond the initial treatment phase, ruminations about the causal attributes of the disease are likely counterproductive for psychological adjustment, and if the “search for meaning” do not lead to “meanings made,” it may amplify distress (Segerstrom et al., 2003; Park et al., 2008). Such a pattern was evident for *n* = 328 long-term breast cancer survivors and a matched control group, in which a continued search for meaning in life >5 years after diagnosis had a negative impact on QoL (Tomich and Helgeson, 2002). Despite of the less consistent association between situational meaning and QoL in general, the ability to find benefits of cancer disease is linked to positive psychological adjustment (Casellas-Grau et al., 2017).

### Incongruence and Meaning-Based Coping

When a discrepancy between global meaning and situational meaning appears, a meaning making process to reduce this discrepancy will be initiated: the positive outcome supporting wellbeing and coping is *meaning made*. The meaning made can take the form of acceptance, “making sense” of the situation and perceptions of growth (Park, 2010). Reports from cancer survivors find occurrence of a cancer diagnosis and treatment to leave a discrepancy between the current life situation and one’s life goals—and over time, meaning making may lead to creating new meanings of the disease, a change in life goals, acceptance and growth (Gall and Cornblat, 2002; Park, 2010; Fallah et al., 2012; Rashidi et al., 2020).

Results of several studies report on both increased meaning (i.e., in relation to others, new conscious way of living), and loss of meaning after cancer (i.e., in relation to relational distress, physical impairment) (Folkman and Greer, 2000; Folkman and Moskowitz, 2000a; Krok and Telka, 2018). In the meaning making process attempting to reduce incongruence between situational meaning and global meaning, the meaning-based coping techniques might be grouped into (1) Changing the meaning of the event (assimilation), and (2) Changing global meaning (accommodation) (Park, 2010; Park and George, 2013). The process of assimilation means that the meaning of the specific situation (i.e., cancer illness) needs to be modified in order to bring congruence to one’s global meaning, and both reattributions and illusions are used to change the situational meaning (Skaggs and Barron, 2006). Attributions and reattributions refer to a continual process of finding the cause of the event, and it is common to look at past behaviors and beliefs in searching for such a cause (Cassel, 1982). Notably, one study found that 42% of survivors of breast cancer (*n* = 322) attribute their illness to be caused by increased levels of psychological stress (Stewart et al., 2001). Attributing the cause of somatic illness to the psyche (i.e., stress) may for many serve as means to regain a sense of control over the illness as the psyche may be perceived as easier to change (Sontag, 1978, pp. 54–56). Such attributions or reattributions may sometimes fall within the categories of illusions, in the sense that they serve as means to regain control of the situation (Skaggs and Barron, 2006).

Becoming overly optimistic and engaging in downward social comparison, where people compare oneself to others who are perceived as worse off, is also common in the assimilation process (Bellizzi et al., 2006). In one of the earlier studies on social comparison among a group of breast cancer patients (*n* = 78), the use of downward social comparison was common (>60% of the women) (Wood et al., 1985). Others have reported similar results where downward social comparison was associated with active coping, seeking social support, reinterpretation of the event and psychological growth (Van der Zee et al., 2000). Patients who suffers from chronic illness, including breast cancer tend to engage in downward social comparison to gain a sense of well-being when they are overwhelmed, unsecure, or feel loss of control over their illness (Terol Cantero et al., 2021). Ascribing new meaning to the cancer illness through assimilation may be beneficial or potentially harmful, and according to the meaning making model, beneficial outcomes are likely when there is a reduction of discrepancy between the new meaning ascribed to the situation and global meaning. In the case of downward comparison, a cancer patient may feel lucky and grateful by comparing oneself to someone who he/she perceives as impaired by illness. Nevertheless, feelings of fear of what might happen to themselves in the future may emerge, thereby increasing the discrepancy to global meaning and cause more distress.

The process of accommodation is initiated when attempts to change the meaning of an event are unsuccessful in reducing incongruence to global meaning (Park and Folkman, 1997). People tend to use different methods to redefine their priorities, set new goals in life, and to revise their belief systems. By applying strategies such as positive reappraisal, problem-focused coping, and revaluing ordinary events, the cancer illness may be viewed in a more positive way.

Positive reappraisal is a cognitive process in which people place greater focus on the good things that have happened to them (Folkman and Moskowitz, 2000b). Positive evaluations of the cancer illness are linked to a positive mood and a more optimistic perspective on one’s health (Sears et al., 2003). One study using a Cognitive Emotion Regulation Questionnaire, found a positive association between cancer patients who perceived their illness as an experience they could learn from, and positive psychological outcomes (Schroevers et al., 2011). A qualitative study of 23 women with breast cancer reported spiritual growth, increased personal strength and appreciation for life as a results of the breast cancer experience (Fallah et al., 2012). For both early stage breast cancer patients and long-time survivors, positive reappraisal is linked to less emotional distress (Sears et al., 2003; Urcuyo et al., 2005), while post-traumatic growth is associated with positive reappraisal for cancer survivors in short and long term (Sears et al., 2003; Widows et al., 2005; Thornton and Perez, 2006; Schroevers and Teo, 2008).

Problem-focused coping involves strategies such as searching for information, problem solving, and the direct actions involved in solving a problem (Folkman and Greer, 2000). Problem-focused strategies may be central in the search for meaning as the focus is on what to prioritize in the situation at hand, evaluating existing goals in life, and setting new goals according to the current situation (Folkman and Greer, 2000; Folkman and Moskowitz, 2000a; Riley and Park, 2014). Although problem-focused coping is important in the early phases of the cancer illness, problem-focused coping may facilitate psychological adjustment even 2 years after diagnosis of breast cancer (Bussell and Naus, 2010). This is in line with conceptualizing cancer to be a chronic illness, “where adjustment is a long-term process of goal adaptation over time” (Naus et al., 2009, p. 64).

Finally, a change in global meaning may also involve increasing the value of events that previously were considered more “ordinary.” This may apply to everyday events such as having dinner, getting together with friends and family, and enjoying nature and beautiful sceneries (Folkman and Moskowitz, 2000a). Despite of increased likelihood of health problems among breast cancer survivors, one Norwegian study reported that the breast cancer survivors were equal to the general population on being physically active (Stalsberg et al., 2019). Furthermore, a recent study of breast cancer survivors find women who place a greater significance on some everyday activities to experience higher levels of QoL (Magnus et al., 2020). As long-term survivors, these women placed more importance on engaging in activities perceived to be good for the body and soul and inspired to creativity, in addition to spending time with family and friends (Magnus et al., 2020).

Many cancer patients report on unmet needs for help with their meaning making processes (van der Spek et al., 2013). Since the efficacy of existential meaning making interventions in cancer patients show positive results in terms of increasing well-being, QoL, hope and self-efficacy, and in reducing depression (Oh and Kim, 2014; Vos et al., 2015; Bauereiss et al., 2018), such interventions have the potential to improve the quality of follow-up care. Based on the empirical literature presented herein, the meaning making process and the outcomes of meanings made depend on the type of stressor, and who is coping with it. It is also common for older individuals to be more oriented toward the past, compared to younger people who are more future oriented (Erikson, 1980). Consequently, age of the person and time since diagnosis are important aspects to consider in studies on meaning making, as the process of meaning making may differ across populations. Furthermore, the majority of previous studies of meaning making among breast cancer survivors are quantitative studies based on questionnaire data (e.g., Sears et al., 2003; Majerníková and Obročníková, 2017; Krok and Telka, 2018). Although studies taking the interpretative phenomenological perspective on meaning making in the population of breast cancer survivors exist, most include data from both young and older survivors (Gall and Cornblat, 2002; Collie et al., 2006; Fallah et al., 2012; Raque-Bogdan et al., 2018; Rashidi et al., 2020). Keeping in mind the increased rate of older cancer survivors in the future, there is a lack of qualitative studies focusing on meaning making in the group of older breast cancer survivors. Therefore, in the current study we aimed to explore meaning making in the lifeworld of older breast cancer survivors using a qualitative approach. By gaining in depth information on the experiences of meaning and meaning discrepancies of this highly homogenous group we aimed to answer the following research question: “How is meaning making shaped between actual situations (situational meaning) and more superior goals and beliefs (global meaning) in older long-term breast cancer survivors?”

## Materials and Methods

### Study Design and Informants

We utilized a qualitative (descriptive and analytic) design involving semi-structured interviews of 23 elderly female breast cancer survivors 7–8 years after their treatment for breast cancer. The interviews followed an interview guide structured along three main themes: “everyday life activities,” “follow-up-care experiences,” and “health status and QoL.” This third theme included both physical, mental, and existential dimensions of QoL.

### Recruitment of Informants

As a part of a larger long-term follow-up study 6–8 years after breast cancer treatment at Trondheim University Hospital, the patients were consecutively invited (by their oncologist or nurse) to participate in an explorative interview-study about life beyond cancer. Patients who agreed to participate were contacted by the last author, and appointments were made for the interviews (place and time). None of the patients received any incentives for participating.

### Research Team and Reflexivity

The interviews were conducted either at the participant’s home or at an arranged meeting place, most often at a dedicated room at the University hospital. The interviews were performed face-to-face by the last and the second author, working within the research fields of psycho-oncology and medical sociology. The remaining authors were engaged in research in the fields of psychology, health informatics, and nursing. The interviews, which lasted for 90 min on average, were electronically recorded, and verbatim transcribed by two independent research assistants. Two researchers analyzed the transcripts, and all subcodes, themes and final categories, including the quotes, were translated into English and thoroughly discussed among all members of the team aiming to strengthen the results’ reliability.

### Data Analyses

The data analysis followed a stepwise, iterative strategy including both inductive, open coding and theoretical discussions, similar to the stepwise-deductive induction approach introduced by Tjora (2018). After transcripts were read thoroughly, we approached the initial data analyses from an inductive perspective, allowing codes to emerge from the data, independent of theory (i.e., the two coders where not familiar with the theory of meaning making at this point in time). In the next step, similar codes were grouped into broader themes. For example, initial codes such as *“life has become harder,” “I do not suffice,” “I am not the same as before,”* and *“I am tired”* were merged into the joint *“life has become completely different.”* All coding in these two steps were conducted using the QSR NVivo 12 software.

The third step of the analysis involved research team discussions of preliminary themes from step two, guided by the theoretical framework of meaning making (Park and Folkman, 1997; Park, 2010). This step was followed by re-categorizations and inclusion of themes fitting the theoretical model. More specifically, we identified themes that reflected the discrepancy between appraised and global meaning, and/or the process in which the informants seemed to attempt reducing this discrepancy (i.e., the meaning making process). As an example, the initial codes *“I am not the same as before,”* and *“I am trying to live as normal”* reflected both a discrepancy between being healthy (normal life before disease) and being tired (normal life after disease), and a tendency of reducing that discrepancy by widening the notion of what includes living a “normal life.” Hence, the final theme *“redefining the limits of normal life”* was established. To resolve any disagreements in coding, and thereby strengthen the validity of the results, the research team met frequently for discussions.

The analyses generated a total of six themes which are presented in the section “Results” with illustrating quotes presented in italics. In order to give a trustworthy representation of the informants’ perspectives, the results are presented with rich descriptions of the categories and several quotes.

## Results

The informants were between 60 and 86 years (mean age = 67 years) at the time of being interviewed. Most of the women were married/cohabited and old-age pensioners. Current or previous occupation was cleaning, administration, health (shorter education) or farming. The women who were still working held executive-, health worker- or management positions. Characteristics of the participants are displayed in Table 1.

**TABLE 1:** Characteristics of the participants (*n* = 23).

**Age (years)**	
Mean (SD) M	67 (7)
Range	60–86
**Marital status, *n* (%)**	
Married / cohabited	14 (61)
Divorced / widowed / single	9 (39)
**Employment status, *n* (%)**	
Old-age pensioners	12 (52)
Disabled	6 (26)
Full or part-time employed	5 (22)

In general, the informants experienced life as quite different from the life before being treated for breast cancer. Many told stories about sleeping problems, pain and fatigue including cognitive and emotional impairments in addition to a sense of emptiness, and a general apprehension of not contributing sufficiently to society. An apt metaphor for returning to ordinary life was that it felt like *jumping on a train in motion.* The interviews also bore evidence of an insoluble conflict potentially arising from incompatible expectations encountered when the women came back to life—cured from cancer and full of desires, but not necessarily being at *good enough* health to live up to neither their own, nor to other’s expectations.

Despite the discernible contrasts between the past and the present, the women seemed to perceive high levels of QoL, focusing on the good things in life rather than getting caught up in trifles or fearing recurrence of cancer. The initial analysis revealed universal and global thematic issues that were perceived as meaningful, such as being healthy, having family and friends, sharing life with a partner, commitment to working, gardening and being outdoors. In addition to these general meaning-categories, descriptions that were more clearly related to having experienced breast cancer, having received treatment, and subsequently the long-term impact on daily life, revealed the more situational meaning categories.

The following text presents the main themes that emerged from the theory-based analysis of the meaning making processes that occurred when the breast cancer survivors attempted to adjust their situational meaning to their overall global meaning system, or vice versa. These themes are more abstract in their construction than the general ones mentioned above, and relate to shifting perspectives and priorities, a strengthened autonomy, widening the limits of normal life, dissociating oneself from the disease, embracing alternative health services, and feeling lucky. The themes are presented with illustrative quotes identified by a pseudonym for each person. All quotes are translated from Norwegian to English.

### Shifting Perspectives and Priorities

Many informants perceived the disease and the following new life situations as a turning point toward a novel awareness that life is not a matter of course, which, in turn, became a reason for living their lives differently and not put things on hold.

*All those things, buy, buy, buy, it’s not important anymore. Rather try to live in presence; it’s better spending your money on nice experiences (rather than things) (May).*

Some of the women even reflected on a possible purpose of the disease, and that the new situation (having experienced cancer) made them change direction in life:

*I believe there’s a meaning to everything that happens. Perhaps it was meant to be, so that I could learn to be more relaxed, blessing in disguise, in a way (Mary).*

Likewise, a more conscious distinction between important and unimportant issues in life was prominent in the material, especially matters that were associated with energy capacity. For many informants, yesterday’s problems had become today’s trifles; *I don’t attach importance to details anymore. When you realize how fragile life can be… Today I really think life is even more valuable. I don’t focus on details anymore (Linda).*

As a result of such changing priorities, there seemed to be a growing awareness of things that previously were taken for granted. It was often referred to as being more aware of their supportive family and close friendships, and of being more present in the very moments. In addition, the women explicitly pronounced a determinate gratification of being able to convey values and important life contents from generation to generation:

*I’m happy for every birthday I celebrate. I’ve grown much more conscious—after having such a diagnosis. I am grateful for the children having managed so well, and I’ve got two grandchildren who will always remember their granny. If I disappear (die), they will always remember me (Elizabeth).*

In sum, the shift in priorities was closely linked both to new ways of thinking, acting and reacting, and in the wake of these alterations, new values were identified.

### Growing Sense of Autonomy

The informants reported that their individual needs and preferences had become increasingly important after experiencing breast cancer. It seemed that the fragility of life had become more visible, resulting in a growing awareness that they must live their life “right now.” Some of the women described it as “an egoism without shame.” Previously in life, they often felt bad when prioritizing their own needs above others’. After having lived through cancer and all its associated experiences, these feelings had changed dramatically. Paying more attention to their own needs and desires and at the same time releasing themselves from bad conscience and shame was a typical reaction observed:

*You must pull yourself together and prioritize your own needs. You can’t sacrifice your own life for others. I try to protect my weekends, use the time on myself. It’s not always feasible, but I try: I feel I need it, need to do pleasurable things—to get my strength back (Linda).*

*Because it has been so busy for so many years, I feel that it’s okay to cool down a bit. If I want to go away for a weekend, I’ll do it, and if I fancy a trip downtown, I’ll do that as well—without getting anyone’s permission… And yes, sometimes I have felt that it was nice having someone visiting me, now and then, but I can decide that myself, it’s my choice (Maria).*

The notion of grasping life events instantly, being more decisive and not wait until tomorrow etc., had become an unambiguously global value among the elderly breast cancer survivors in this study.

*I have become very stingy with my time. I don’t care about so many things… I hear people saying, “you must go there,” and “you should do that”… I live here, among my closest family… Why should I go to Mexico? I don’t see the point (Margaret).*

These shifts in global values did not happen rapidly. Rather, they required specific actions (from the women) to reach there. Many of the breast cancer survivors described their everyday life quite different from the life they lived before the cancer (and in their younger days), especially in terms of lack of energy. The breast cancer survivors felt they were not able to do things that they used to do before the illness. For example, many of them expressed that it was too demanding to invite family and friends for dinner, to join interest groups, or to make any long-term plans. The women told how they actively decided how and with whom they spent their time and how they were “forced” to reorganize their lives to redistribute their energy to live meaningful lives:

*I held positions in several organizations, and surely, I fulfilled my obligations. Now I have reduced such activities. You turn more egoistic and self-centered, and you think; No, I don’t want to bite off more than I can chew (Lisa).*

*People (friends) call me, asking me to join them at different events, but I have become so stubborn and weird; I hide, and I only join things that suit me. My friends must humor me. In earlier days (before) I was amenable, but nowadays I’m not, I become easily irritated/annoyed (Karen).*

The discrepancy or experienced gap between the previous “shame over being selfish” and the current “proud of taking own needs seriously” could be interpreted as a growing sense of autonomy and be the outcome of this meaning making process.

### Widening the Limits of Normality

Redefining the notion of “a normal life” emerged as another weighty theme in our material. Many reported having a good health, even though they struggled with several health issues. Being healthy was likely a global desire and perceived important to be associated with. Being an ill and weak person was difficult to adapt to. The women seemed to be reducing the discrepancy between the self-perception of being a healthy human and the current feeling of being impaired, by widening the limits of what defines a normal life. Almost all informants explained their current health problems, such as pain, reduced cognition, or physical capacity (the situation) to be a consequence of growing older rather than resulting from late effects of treatment. Typical thoughts were:

*It happens a lot to your body when you get cancer, and all you’ve been through …It’s difficult to know whether they (the problems) are due to the disease, all you’ve been through, or only a result of aging. I don’t know (Mary).*

At the same time as they accepted the changes, some of the women emphasized that even managing everyday tasks, such as making dinner, gave evidence of living as normal:

*I try to live as normal as possible […] Yes, I do—I make dinner and all… I’m living a quite normal life now, so I have returned to the good old me…even if it’s…you know…you’ll never be the same. I just have to take my time…(May).*

Redefining the notion of normality became apparent likely because the breast cancer survivors strongly wished to appear as active members of society, even though many were both physically and cognitively impaired.

### Dissociating Oneself From the Disease

An opposite strategy to widening the limits of normality was the denial, or at least striving to keep distance to the disease and anything that could be associated with it. Such strategy was often materialized by avoiding any participation in breast cancer supporting groups, as described by this woman:

*I don’t want to attend such a (BC-support) group. I don’t want to talk about disease. I don’t want to keep company with people with cancer. I want to lift me up—want to be with people who don’t talk about disease (Karen).*

Another way of dissociating oneself from the cancer disease, was to make no place for breast cancer in their lives, as quoted: *I don’t think about it. I don’t worry or speculate. In fact, I pretend to be healthy (Linda).*

Dissociating oneself from illness and patient experiences, simply by defining oneself not belonging to the patient group, seemed decisive for many of the women in maintaining the important perception of being connected to society.

### Embracing Alternative Health Services

Many of the survivors were struggling with various health problems, which were ascribed to either late effects of breast cancer treatment, or as a part of getting older. Even though most of the women accepted that their health and daily life were totally different compared to the situation prior to their breast cancer history, some of them praised different alternative and self-initiated ways of improving their health and thereby the quality of everyday life. For example, one of the informants reflected a deep interest in yoga and mindfulness and seemingly believed in the positive effect of these activities for improving sleeping quality and pain relief:

*I must admit, there have been some bodily alterations, both physically and mentally. I have attended some courses in mindfulness that I found very helpful. I easily fell asleep every evening afterward. I also decided to start with yoga—it is once a week. I found that I needed to stretch the muscles—I feel that it helps for the pain in my arm—I have a lymph node removed… (Mary).*

Another informant talked enthusiastically about the health-related impact of healing methods/techniques:

*I believe it is more to heaven and earth. Our travel guide practiced a Japanese treatment technique called Reiki. It is a kind of energy transference…how should I explain? You get more energy, better quality of life, better sleep, and such things. I had one “treatment,” and I’ve never felt so relaxed in all my life. It was very strange… Later she arranged courses, and I attended with several others, and it was useful/instructive (Maria).*

The positive effect of homeopathy and acupuncture were mentioned by some informants, especially the benefits related to better sleeping quality and increased physical surplus:

*One of my colleagues had good experience with acupuncture. So, I have spent 4000 (NOK), no matter whether it works or not, but I’ve got better. Although I’m still not completely healthy, I manage to sleep better at nights (Sandra).*

*I had reached rock bottom when I returned to work. I went to a homeopath who offered acupuncture, and that got me on top. I visited him for a year. I think that was the reason why I manage to work after the cancer treatment. I had a great confidence in homeopathy, and my general practitioner agreed with me (Helen).*

Lastly, some of the breast cancer survivors also stated a firm belief that dietary supplements could be useful in improving the level of energy. One woman expressed the fortune of knowing someone who could provide these health promoting remedies, as follows:

*I have my own methods, you know, through acquaintances, I’ve got some tips. Last year I heard about something special, expensive though! Something named “Synergy”—a lot of vitamins and minerals and other ingredients. I have used it since July last year and I feel a great difference. It works very good for me. I‘ve got a quite different everyday life since I started (Ruth).*

### Feeling Lucky

Luck was a recurrent construct in our material. Even though the women had been through a breast cancer diagnosis, subsequent treatment accompanied by anxiety and worries, and still were experiencing bothersome late effects; almost all of them stated -in one way or another- how lucky they felt after all. The luck they explained was associated with various aspects in life; from the most fundamental aspect of having survived to the gratitude over different life events, which they previously had taken for granted. Many of the breast cancer survivors reported that all in all, they had been very lucky compared to others—whether it was someone they knew or other persons they had heard about. The consequences of the disease could have been so much worse, such as loss of life, worse adaption to the treatment, spread of cancer, or a myriad of late effects. The feeling of luck was extensively expressed by the breast cancer survivors:

*I’m praising every new day I get, and I don’t understand why I should be the one who survived…, because… they died like flies around me (Sarah).*

*Afterward, when I reflected on what I’ve been through, I thought that I’ve really been lucky, because the cancer hadn’t spread to other organs (Elise).*

*I’m lucky for being as healthy as I am. I know so many people who had a relapse of the cancer, and suddenly they’re gone. So, I feel lucky to be so content with my life (Lucy)!*

Although some of the women were striving with both late effects of treatment, other comorbidities, and even challenging family situations, they still adopted a perspective of luck. The women explained their luck or gratitude in several ways:

*My breast is deformed, and I find it disgusting, but I don’t care. That’s how it turned out, and I’m lucky who got cured (Evelyn).*

*I’ve got the best treatment and I’ve been strong enough to tolerate it, so I‘ve been very lucky (Mary).*

## Discussion

Guided by the meaning making model developed by Park (2013), the present study aimed to explore the meaning making in the lifeworld of older breast cancer survivors, with a main focus on exploring discrepancies between situational and global meaning. As expected, the contrast between past and present was a highlighted theme in the interviews. Based on analyses of the women’s narratives, the process of meaning making and efforts to adjust discrepancies between situational and global meaning were grouped into six main themes; (1) shifting perspectives and priorities, (2) growing sense of autonomy, (3) widening the limits of normal life, (4) dissociating oneself from the disease, (5) embracing alternative health services, and (6) feeling lucky. However, as the third and the fourth theme both relates to an adjustment to normal life, they are discussed under the same heading.

### Shifting Perspectives and Priorities

The general finding that the women perceived a shift from before the cancer illness were expressed as major turning points in life. Sudden and profound changes in global meaning was reported as a direct cause of the illness. Some came to see the cancer experience as a blessing—the meaning of cancer illness was to facilitate a stronger focus on what they considered important in life. Such positive appraisals of the cancer illness are linked to a reduction of incongruence to global meaning (Park and Folkman, 1997). For the women reported on here, a revision of their belief system was common. They became more conscious about how they spent money and their time, and many issues they considered important before were now considered trivial. Furthermore, by revaluing ordinary events, the women’s global meaning shifted toward generativity, which includes individual (and societal) goals of providing for the next generation (McAdams and de St Aubin, 1992); they expressed gratitude for being able to convey values and important life contents to children and grandchildren, and the value of a supporting family and friends were highlighted. All in all, the women used different coping strategies (i.e., positive reappraisal, problem-focused coping, revaluing ordinary events) to view the cancer illness in a more positive light, accommodating a shift in global meaning. The ability to transform the meaning of a traumatic experience to a positive experience is associated with the outcomes of a more positive mood, optimistic view on one’s health, less emotional distress, and post-traumatic growth (Sears et al., 2003; Urcuyo et al., 2005; Widows et al., 2005; Thornton and Perez, 2006; Schroevers and Teo, 2008; Schroevers et al., 2011). A qualitative study of 11 breast cancer survivors aged 40–86 years reported what they termed “identity transformation” which was a turning point toward promoting positive meaning making, empowerment, and control. The profound changes in global meaning after the cancer disease were described as a sudden shift toward living a more authentic life (Rashidi et al., 2020). In our study, the women’s sudden shift in global meaning was experienced as a positive turning point in life, which is in line with the evidence of meaning in life as a buffer against distress and a promotor for well-being and QoL (Tomich and Helgeson, 2002; Winger et al., 2016; Majerníková and Obročníková, 2017).

### Growing Sense of Autonomy

The shift toward greater levels of autonomy can be conceptualized as accommodations of global meaning (Park and Folkman, 1997). From the cancer experience the women became more aware of the fundamental existential fact that life is not infinite. Both a stricter practice and an expansion of own boundaries grew from this new perspective on life. This involved making an autonomic choice of “no action” in situations they previously had acted (i.e., stop having large dinner parties)—and “acting” when they previously did not (i.e., travel and engage in activities without asking “permission” from others). Some women viewed the cancer experience as a blessing that made them act in accordance with what was most important in life. These findings are in line with the process of positive reappraisals of the cancer disease (Folkman and Moskowitz, 2000b), which is linked to well-being and optimistic perspectives on own health (Sears et al., 2003). All in all, the women in our study were able to transform meaning discrepancies into a new global meaning boosting autonomy.

The ability to feel a full sense of ownership to one’s behavior, regardless of traumatic life event also develops with age (Sheldon et al., 2006). According to self-determination theory people have an innate need to seek out autonomy because it is a universal inherent psychological need—and as people grow older they might find new ways to take control over their lives (Deci and Ryan, 2000). Non-internalized parts of oneself will with time be assimilated into a coherent whole (Deci and Ryan, 1991). Indeed, one study revealed that in the case of younger cancer patients a more intense search for meaning was more common compared to older patients (Schroevers et al., 2004). On the other hand, with age follows a general trend of cognitive decline and a higher likelihood of physical impairment (Ferraro and Carr, 2021), that may for some reduce the experience of autonomy (Sánchez-García et al., 2019). For the older women in the current study lack of autonomy was not expressed, even though these women report on several mental and physical health complaints. Thus, the increase of autonomy following cancer survivorship (for the women in the study) could (to some degree) be linked to their age and their likelihood of knowing themselves more. On the other hand, older age in combination with the cancer experience could cause the development of a stronger autonomy as trauma affects peoples evaluations of their capabilities, in a positive way, to face life problems in the future (Lelorain et al., 2010).

### Widening the Limits of Normal Life and Dissociating Oneself From the Disease

Despite physical and cognitive impairments, being normal was important. The women defined normality as the ability to engage in the same everyday activities as before the cancer. Furthermore, a sense of normality was maintained by explaining physical and cognitive challenges to be a result of aging—a “normal” process—as opposed to a consequence of the disease, which is unexpected and generally less likely to occur. Maintaining age as an explanation of health problems may facilitate a feeling of being normal, and of having more control of the situation.

Similar to the present findings, one qualitative study of a mixed group of cancer survivors (*n* = 40) reported that upholding a more or less unchanged everyday life was important to them in life after cancer treatment (Henshall et al., 2016). A goal for the informants who expressed a “restitution illness narrative” was to do everything as before, including hobbies, travels, and other activities. However, somewhat contrasting to the findings presented here was that the informants presented themselves outward as if all were normal (when it was not), motivated by not wanting to be labeled, or being a burden to others (Henshall et al., 2016). Another group of informants in Henshall et al. (2016) study reported on a “quest illness narrative” which was more in line with findings of the present study where survivors had accepted that they needed to learn how to live with the cancer, which is more in line with a definition of normality as accommodations of global meaning. Although the women in the present study held on to their image of having good health and being active members of society, it appeared that they had become more flexible in terms of what it means to be “healthy” and “active” after the cancer.

As the present findings support an adjustment of global meaning through developing new meanings of “normality,” the women also explained that they did not want to associate themselves with the disease and refrained from e.g., participating in cancer support groups. These two strategies may both be viewed as a form of denial with the goal of preserving some level of normality –accommodation through expanding the notion of normality, and assimilation through distancing from the disease. The paradox of denial coping is that it may affect the psychological functioning in either a beneficial or detrimental way depending on active versus passive denial, respectively (Vos and De Haes, 2007). Positive psychological outcomes of denial are reported for distractive strategies that have the function of facilitating a positive outlook on life. The more passive escape strategies, on the other hand, is related to poorer psychological functioning (Goyal et al., 2018). It is evident that the women reported on here show a preponderance of active mobilization toward something positive, while denying the illness. In this way, they expand the boundaries of normality, accommodating global meaning.

A review of several longitudinal studies of patients with various types of cancer with study periods varying from 6 months to 3–5 years after diagnosis reported that denial coping diminished with time. On the other hand, the review found that when faced with death, an increase in denial coping became evident (Vos and De Haes, 2007). Indeed, a qualitative study of a mixed group of older cancer survivors (>65 years) reported that the cancer disease in the context of the aging body and decline became rather normalized. Approaching death was a theme for the informants, but as a consequence of age and not cancer (Hannum et al., 2016). Relative to other problems in old age, the potential detrimental consequences of cancer disease were denied by the informants. In line with the findings reported here, denial served for the most part as an adaptive coping strategy facilitating well-being and QoL (Hannum et al., 2016).

### Embracing Alternative Health Services

The women were engaged in various complementary and alternative medicine (CAM), trying to ease or eliminate poor mental and physical health to improve their QoL such as practicing yoga and mindfulness, use of healing techniques, homeopathy, acupuncture and taking dietary supplements. A previous study reported that 59% of middle age- and older long-term breast cancer survivors use CAM, and these women had a higher prevalence of co-morbidities and poorer emotional functioning (Carpenter et al., 2009). The reasons for engaging in CAM is linked to the concept of health empowerment which is the ability of a person to use the necessary resources in order to achieve an improvement in one’s health (Bann et al., 2010). Indeed, among cancer patients the use of CAM could be motivated toward reducing feelings of helplessness, to be actively engaged in- and to increase control over own health. The use of CAM may therefore be an active and problem focused coping strategy to prevent and relieve physical and mental problems after cancer illness. This is in line with the findings of the present study as the women report that their own initiatives to CAM activities relieve pain, improve sleep quality, and increase energy. The outcome of assimilation and accommodation in “meanings made” for situational and global meaning was therefore quite common for the breast cancer survivors. The women have developed their understanding of the causal relationships between the cancer illness (consequences of the disease) and health outcomes. Placing themselves as active agents able to reduce detrimental consequences of the disease with CAM is a way of empowering themselves and giving new meaning to the illness. Furthermore, the women in our study shared that they also experienced new global meaning—that the engagement in CAM had altered their outlook and perspective in life. Engagement in CAM was an eye-opener to the life ahead, and they got new perspectives on their everyday life. Similar to our findings, a previous study on benefit-finding after cancer experience, reported that the use of some types of CAM was associated with finding benefit from the illness regarding personal priorities, daily activities and family as a result of changed attitudes and behavior caused by their experience of cancer (Garland et al., 2013).

### Feeling Lucky

The current study reported that feelings of luck and gratitude thinking about current life situation was a common theme among the breast cancer survivors. The women reflected on the increased risk for losing their lives to cancer, complications, and late effects of the cancer disease. Comparing themselves with others, including fictive persons, who were perceived as worse off, was common. Such coping methods of downward social comparison is common for assimilating new meaning to an adverse situation (Van der Zee et al., 2000; Bellizzi et al., 2006). The dichotomy sick versus healthy may for the women reflect the discrepancies between situational and global meaning. They conceptualized their own situation to be closer to the “healthy” end of the dichotomy. This adjustment was done by assimilating new meaning to the cancer, the meaning of feeling lucky. Through these mechanisms, the women were able to see the cancer illness and themselves in a more positive light. Downward social comparison may promote QoL for cancer patients, and is associated with active coping styles such as reinterpretations and seeking social support (Van der Zee et al., 2000). However, several studies report that health promoting mechanisms related to downward social comparison depend on identification vs. contrast processes (Van der Zee et al., 2000; Brakel et al., 2012a, b; Buunk et al., 2012). That is, positive outcomes of downward social comparison is more likely when the person identifies with, as opposed to feel very different from (e.g., situation, personality etc.) the comparable person (Van der Zee et al., 2000). In the current study we do not tap into the level of strong or weak identification with the comparable persons the women refer to, but the common experience of breast cancer could increase feelings of identifying with other cancer survivors. Breast cancer is a women’s disease in which most are diagnosed from the mid-40s and up. Furthermore, international establishments of “breast cancer culture” in the public also comes with a set of attitudes and values that may facilitate connectedness to others with cancer (Bell, 2014). Consequently, it is likely that many women strongly empathize with others who have experienced breast cancer. Furthermore, the time since diagnosis may also influence on the beneficial versus negative result of downward social comparison; in early stages of diagnosis and treatment, you may look at others who are worse off and fear what awaits you when the disease advances. The long-term survivors in the current study is more likely to have a distance to the initial treatment and they have a more stable situation in which recurrence of the disease is likely smaller. Therefore, feeling lucky as “meanings made” to the cancer illness reduce the gap between situational and global meaning, and seem beneficial for increased well-being and QoL.

## Conclusion

This study provided an in-depth exploration of meaning and meaning discrepancies of older breast cancer survivors 7–8 years after diagnosis. The women struggled with several health problems in the aftermaths of the disease, such as sleeping problems, pain, and fatigue—including cognitive and emotional impairments. Meaning and meaning discrepancies was concentrated on six main themes: shifting perspectives and priorities, growing sense of autonomy, widening the limits of normal life, dissociating oneself from the disease, embracing alternative health services, and feeling lucky. In light of the meaning making model by Park (2013), a wide range of coping techniques were initiated to change global meaning in order to transform the cancer disease into a more positive experience. Common coping efforts across the six main themes were social comparison, denial, positive reappraisal, problem-focused coping, and revaluing ordinary events. Through the process of accommodation involving different coping strategies the women experienced a comprehensive shift in their global meaning, sorting out and acting on the important things in life. The women became more respectful for their own needs through increased autonomy, and expressed kindness toward themselves with redefining what before the disease was considered a “normal” way of living. In the assimilation process, social comparison was common as they viewed their own personal experience with cancer disease as easier compared to others, and therefore they expressed luckiness. Denial coping were used to distance themselves from the cancer disease, distracting themselves and focusing on the positive. Attributing possible cancer-related late effects to age was also pronounced. Although cancer disease may lose significance among older survivors due to increased comorbidities as health problems becomes the norm (Hannum et al., 2016), this was not reported here. Problem-focused coping was also common as they, among other initiatives, sought aid in CAM. Not so common was reattributions of the cause of the cancer disease. In general, the women had come to terms with, and accepted their destiny. In case of attributions, the disease was viewed as having higher purpose, bringing positive changes to their lives. In comparison, younger cancer survivors tend to struggle more, and searching more intensely for the purpose of the disease (Schroevers et al., 2004). In conclusion, a rich pool of coping efforts, often complementing each other, were used by these women to decrease the discrepancy between global and situational meaning.

The study’s strengths centered on the homogenous sample of older (60–87 years) breast cancer survivors, all being equal in time since cancer treatment (7–8 years) which allows for generalizing the findings to older, long-term survivors of breast cancer. On the other hand, the long timeline could result in recall bias and thereby influence the study’s reliability. The interviews were conducted to gain knowledge on life beyond cancer, and the semi-structured interviews were not specifically designed for gaining in depth information on meaning making. Although the number of interviews (*n* = 23) provided rich material, and the process of analyzing the data was comprehensive due to the introduction of the meaning making model halfway in the analysis, the interviewer could not at this point ask the informants to clarify or invite the women to further explore themes relevant specifically for meaning making. Future studies should therefore focus specifically on meaning and meaning making. Furthermore, some additional constructs evinced significant for coping and meaning making such as self-compassion and emotional intelligence should be included to get a deeper understanding of the phenomena of the meaning making process for older breast cancer survivors (Teques et al., 2016; Ewert et al., 2021). Finally, future research should aim for conducting longitudinal qualitative research on meaning making for gaining knowledge on meaning making trajectories from onset of the cancer illness, to long-term survivorship. This is especially valuable given that cancer survivorship is a process of living after a diagnosis—and lasts throughout life (Mullan, 1985).

Meta-analyses provide evidence that existential meaning making interventions are effective and helpful for patients across all stages and types of cancer (Oh and Kim, 2014; Vos et al., 2015; Bauereiss et al., 2018). They are found to enhance perceived meaning in life, well-being, and mental health, and to reduce psychological distress after cancer (Henry et al., 2010; van der Spek et al., 2017). Like the findings of the current study, meaning making efforts can be successful as the life for many survivors become more meaningful after cancer disease. Contrary, many cancer patients report on unmet needs for help with their meaning making (van der Spek et al., 2013), and the facilitation of meaning making processes is rarely included in the follow-up care for cancer survivors (Selby et al., 2017). Thus, the practical implications from the current study may help health care professionals provide care for women who have experienced breast cancer. The concrete knowledge of common coping efforts in the meaning making process may help in developing future interventions and gaining a deeper understanding for older survivors of breast cancer.

## Data Availability Statement

The datasets presented in this article are not readily available because participant consent don’t allow to share data with third parties. Requests to access the datasets should be directed to marn@ntnu.no.

## Ethics Statement

The studies involving human participants were reviewed and approved by Regional Committee for Medical and Health Research Ethics (REC Central 2009/108.4.2006.2856). The patients/participants provided their written informed consent to participate in this study.

## Author Contributions

RS, KS, GH, MN, and RJR made substantial contributions to the study, including conceptualization, design, methodology, and analyses. RS, KS, and RJR were mainly performed the data analyses. MN did mainly perform the introduction, discussion, and conclusion. RS and RJR wrote the methods and results. All authors reviewed and edited the manuscript and approved the final version of the manuscript.

## Conflict of Interest

The authors declare that the research was conducted in the absence of any commercial or financial relationships that could be construed as a potential conflict of interest.

## Publisher’s Note

All claims expressed in this article are solely those of the authors and do not necessarily represent those of their affiliated organizations, or those of the publisher, the editors and the reviewers. Any product that may be evaluated in this article, or claim that may be made by its manufacturer, is not guaranteed or endorsed by the publisher.
